# Supplementary material for: H1N1 Influenza A Virus Protein NS2 Inhibits Innate Immune Response by Targeting IRF7
Source: Viruses. 2022 Oct 31;14(11):2411. doi: 10.3390/v14112411 (PMC9694023; doi:10.3390/v14112411)
Supplement: Supplementary file 1 [file viruses-14-02411-s001.zip › viruses-1977020-supplementary.pdf]

**Supplementary Materials:**

**Table S1.** PCR primers used in this study.

| Primer name | Primer sequence |
|---|---|
| IFNB1-Forward | GCTTGGATTCCTACAAAGAAGCA |
| IFNB1-Reverse | ATAGATGGTCAATGCGGCGTC |
| ISG15-Forward | AGGACAGGGTCCCCCTTGCC |
| ISG15-Reverse | CCTCCAGCCCGCTCACTTGC |
| ISG56-Forward | TCATCAGGTCAAGGATAGTC |
| ISG56-Reverse | CACACTGTATTTGGTGTCTAGG |
| IFIT2-Forward | AAGCACCTCAAAGGGCAAAAC |
| IFIT2-Reverse | TCGGCCCATGTGATAGTAGAC |
| RANTES -Forward | GGCAGCCCTCGCTGTCATCC |
| RANTES -Reverse | GCAGCAGGGTGTGGTGTCCG |
| GAPDH-Forward | TCATTGACCTGAACTACAT |
| GAPDH-Reverse | GAAGATGGTGATGGACTT |
